# Nursing homes and the elderly regarding the COVID-19 pandemic: situation report from Hungary

**DOI:** 10.1007/s11357-020-00195-z

**Published:** 2020-05-18

**Authors:** Gábor Kemenesi, László Kornya, Gábor Endre Tóth, Kornélia Kurucz, Safia Zeghbib, Balázs A. Somogyi, Viktor Zöldi, Péter Urbán, Róbert Herczeg, Ferenc Jakab

**Affiliations:** 1 Virological Research Group, University of Pécs, Szentágothai Research Center, Pecs, Hungary; 2 Faculty of Sciences, Department of Genetics and Molecular Biology, University of Pécs, Pecs, Hungary; 3 University of Pécs, National Coronavirus Research Center, Pecs, Hungary; 4 Central Hospital of Southern Pest – National Institute of Hematology and Infectious Diseases, Budapest, Hungary; 5 Faculty of Sciences, Department of Ecology, University of Pécs, Pecs, Hungary; 6 Vantaa, Finland; 7 Bioinformatics Research Group, Genomic and Bioinformatics Core Facility, University of Pécs, Szentágothai Research Center, Pecs, Hungary

**Keywords:** nCoV2019, Outbreak, Genome sequencing, Hotspot, Coronavirus

## Abstract

The global impact of the severe acute respiratory syndrome coronavirus 2 (SARS-CoV-2) pandemic is significant in terms of public health effects and its long-term socio-economic implications. Among all social groups, the elderly is by far the most affected age group regarding morbidity and mortality. In multiple countries spanning several continents, there are an increasing number of reports referencing the novel coronavirus disease-2019 (COVID-19) spread among nursing homes. These areas are now recognized as potent hotspots regarding the pandemic, which one considers with special regard. Herein, we present currently available data of fatal COVID-19 cases throughout Hungary, along with the analysis of the co-morbidity network. We also report on viral genomic data originating from a nursing home resident. The genomic data was used for viral haplotype network analysis. We emphasize the urgent need for public health authorities to focus on nursing homes and residential service units worldwide, especially in the care of the elderly and infirmed. Our results further emphasize the recent statement released by the World Health Organization (WHO) regarding the vulnerability among seniors and especially the high risk of COVID-19 emergence throughout nursing and social homes.

## Introduction

As a consequence of our rapidly changing and massively globalized world, the dynamics and extension of outbreaks also change. Although humanity has already faced a number of pandemics in the twentieth century, the virus SARS-CoV-2 (causing COVID-19) represents one of the biggest challenges the globalized world has yet confronted. Notably, as the situation evolves worldwide surpassing 2 million cases, there are important aspects to consider. The vulnerability among individuals with underlying medical conditions and specific social groups is now evident, as there are numerous nursing homes around the world currently heavily affected by COVID-19 (Davidson and Szanton 2020; Bedford et al. 2020).

Whereas COVID-19 is a new disease and there is limited information regarding risk factors for severe disease, based on currently available epidemiological data from multiple continents, it is clear that the elderly population is at a significantly higher risk of severe or fatal outcome of COVID-19. However, all age groups are at risk of contracting COVID-19, and seniors face a significant risk in developing a severe illness if they contract the disease due to physiological changes which come with aging and more frequently, underlying health conditions in this particular age group. Recent epidemiological data from Wuhan suggests 5.1 times higher risk of dying in symptomatic elderly patients aged 60 years and above (Wu et al. 2020), and overall, over 95% of these deaths occurred in those older than 60 years as recent WHO data. Similar to several other European countries, the present Hungarian population shows an aging population pyramid, approximately 19% of the Hungarian population are older than 64 years, or 1.8 million people (HCSO 2020, Fig. 1). The very first reported COVID-19 case in Hungary was confirmed on the 3rd of March 2020. During the first 7 weeks of the epidemic, we reached the first 100 fatalities.

**Fig. 1:**
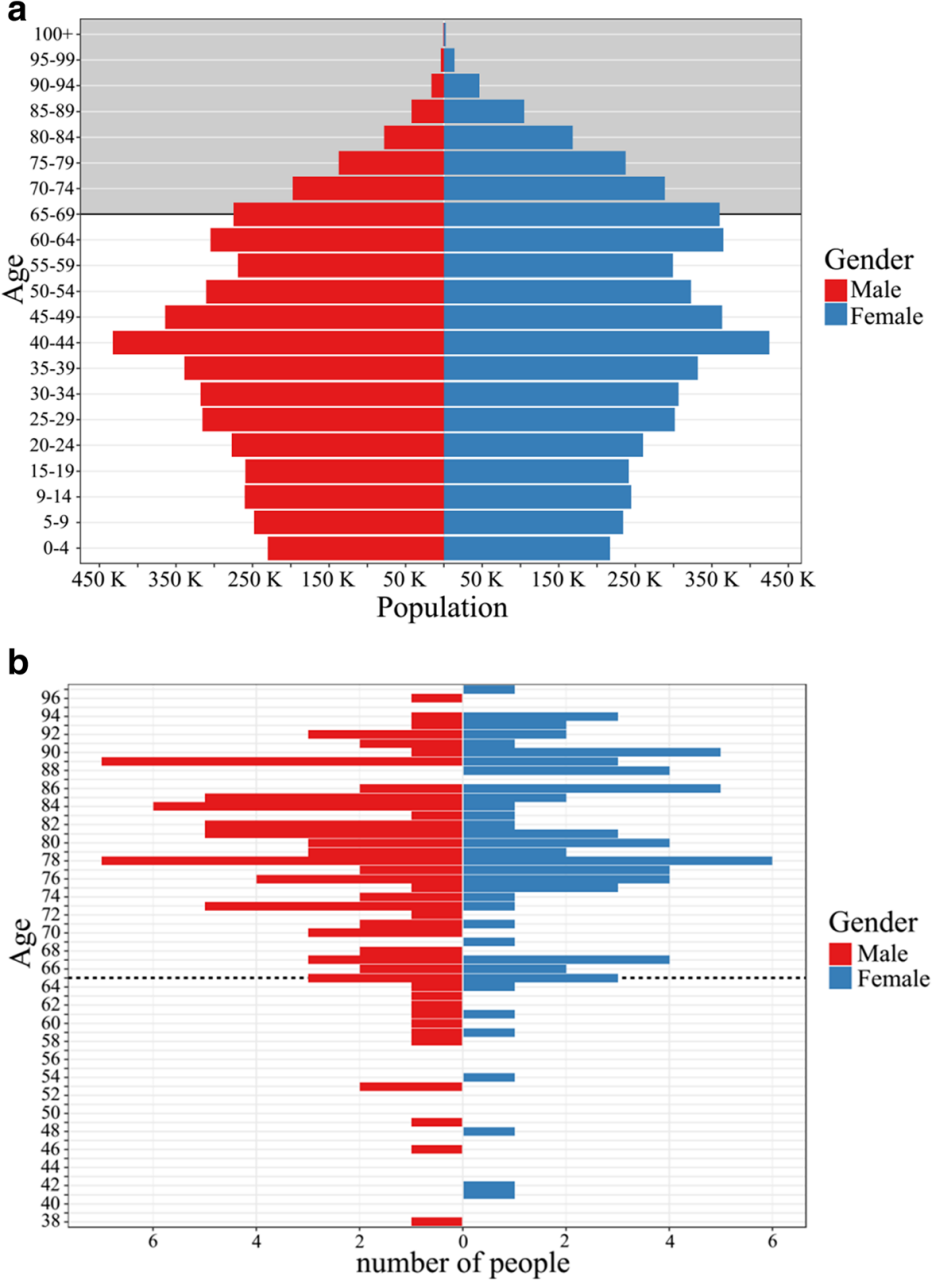
**a** Age distribution (age-gender pyramid) of the Hungarian population (red: male, blue: female); based on data from the Hungarian Central Statistical Office. Gray background highlights the elderly section (individuals aged 65 years or older) of the population. **b** Age distribution of fatal cases (red: male, blue: female) with confirmed COVID-19 infection in Hungary as of 18 April 2020

Additionally, individuals of any age with underlying medical conditions, particularly if unrecognized or not well controlled, may likely be at a higher risk regarding severe illness from SARS-CoV-2. Moreover, the prevalence of comorbidities may be a further risk factor for a severe outcome (Yang et al. 2020). Based on WHO statement from the 2nd of April, 8 out of 10 deaths are occurring among individuals with at least one comorbidity (particularly cardiovascular disease, hypertension, and diabetes) (WHO 2020).

It was noted prior to the current pandemic, elderly populations living in nursing or retirement homes have predominant roles in epidemics, since circumstances in a retirement home provide an ideal environment for the acquisition and spread of infection: susceptible residents who share sources of air, food, water, and health care in a crowded institutionalized setting. Moreover, the constant movement of visitors, staff, and residents establishes a constant and complex transmission route with the outside community. Among others, outbreaks of respiratory and gastrointestinal infection predominate in this setting, e.g., influenza, rhinovirus, and norovirus infections (Juthani-Mehta and Quagliarello 2010). Today, Hungary has nearly 1100 active nursery homes with approximately 55,000 residents (HCSO 2020), which is a significant risk factor for outbreaks, and should be prioritized by public health authorities.

In this paper, we summarize the epidemiological data regarding COVID-19 in connection to the elderly population in Hungary. We summarize and analyze the clinical details of fatal cases and offer a special emphasis on the role of retirement homes in the current pandemic. We also present the phylogenetic analysis of a nursing home-derived virus sequence.

## Materials and methods

Epidemiological data were collected from the database of the Hungarian Academy of Sciences (https://radex.hu/covid/) dedicated in the follow-up of the Hungarian COVID-19 situation based on all available official government sources.

A SARS-CoV-2-positive sample was obtained for analysis from a dedicated diagnostic laboratory, following confirmation as a positive case, and the ensuing official diagnostic chain. A nucleic acid sample was extracted directly from oro-pharyngeal swab samples using a Direct-zol™ RNA MiniPrep Plus extraction kit (Zymo Research), in compliance with the manufacturers’ recommended guidance.

We performed a reverse transcription using SARS-CoV-2 specific and random primers. The library was prepared by NEBNext Ultra II RNA Library Prep Kit for Illumina (New England Biolabs). Both the concentration and the fragment size of the library were measured and checked with the Agilent TapeStation 4200 (Agilent Technologies) and Qubit 3.0 Fluorometer (Thermo Fisher Scientific). Library preparation was followed by sequencing on Illumina iSeq 100 with 2 × 150 bp read length (Illumina).

Raw sequences were quality checked with FastQC (Andrews 2010). During the next step, the readouts were trimmed with Trimmomatic (Bolger et al. 2014). The remaining high-quality readings were mapped against the MN908947 reference sequence. For the mapping, Geneious (Geneious Prime 2020.0.3) default algorithm was applied with the default parameters. A consensus sequence was created by Genenious consensus calling algorithm, in which the minimum coverage was set to 15.

To understand the structures of the symptoms’ common occurrence, the interactions between causes of deaths were analyzed with network analysis. The co-morbidity data were visualized in a R environment (R Core Team 2020) with *igraph* package (Csardi and Nepusz 2006).

In order to construct a haplotype network analysis from the sequence dataset, the median-joining network method implemented in popart software was used (Bandelt et al. 1999). Sequences were aligned in MAFFT with default settings, converted to nexus format and subsequently modified in accordance with the popart example file. Sites with missing nucleotides were excluded from the analyses and the value of the epsilon was set to zero. Sequence from the current work (*SARS-CoV-2/Budapest/303/202004*) and three additional Hungarian sequences were included from our country into the analysis (derived from GISAID database which were available on 21 April 2020).

## Results

As of 18 April 2020, the total number of laboratory-confirmed COVID-19 cases in Hungary was 1834. Among those, 1431 were active cases, 231 were recovered, and 172 deaths (95 males and 77 females, 55 and 45%, respectively) occurred. Overall, two-thirds of the cases were aged 50 years and above. The distribution of cases among age groups was the following: individuals aged 70 years or older—35%; between 50 and 69 years—30%; between 30 and 49 years—23%; between 15 and 29 years—10%; children aged 14 years or younger—2%. The age distribution among deaths due to COVID-19 is depicted in Fig. 1b. The age range of fatalities was 38–97 years with a mean of 77 years and median of 78.5 years. Overall, 93% of the deaths occurred among cases in the 60+ age group.

**Fig. 2:**
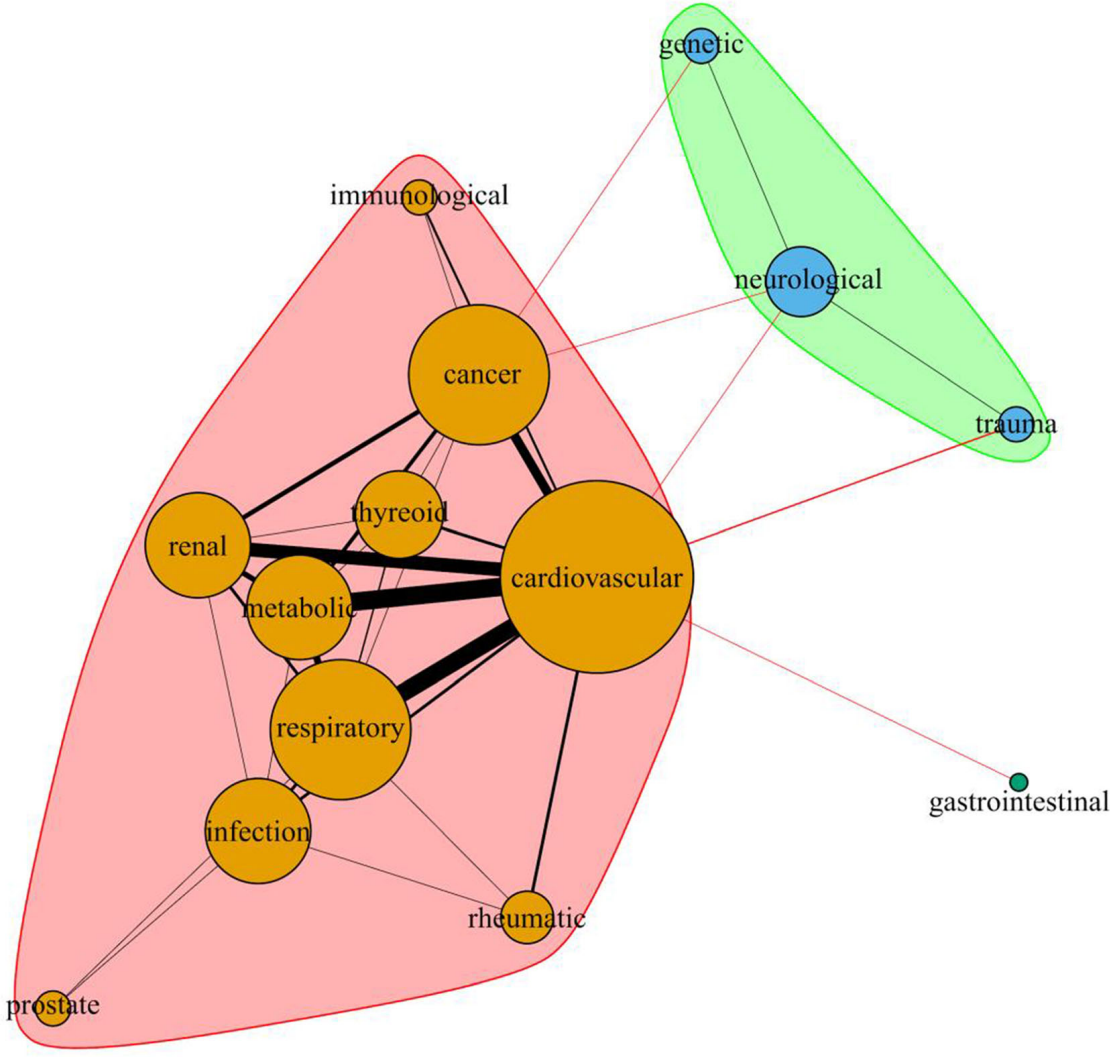
Network of the underlying health conditions and frequency of comorbidities in fatal COVID-19 cases in Hungary (*N* = 172). The bigger size of the bubble represents the higher number of cases, and the bolder line represents the more common comorbidities (more frequent co-occurrence of different diseases or disorders)

However, in 14 cases, there was no data available regarding the general health status of the patients, most of which had known underlying diseases. In the majority of fatal cases (132, 76.7%), the patients had cardiovascular diseases (including heart rhythm disorder, hypertension, coronary artery disease, atrial fibrillation, aortic stenosis, rheumatic heart disease, cerebrovascular problem, arteriosclerosis, and stroke); however, in the majority (57.5% of deaths), it was associated with further health problems, particularly with metabolic disorders (including diabetes mellitus and hypo- or hyperthyroidism) and/or respiratory diseases, chronic renal failure, different kind of tumors, and even gastrointestinal disorder or rheumatic problems in some cases, and various combinations of all of these (Fig. 2). Respiratory disorder, namely chronic obstructive pulmonary disease (COPD), was mentioned in 21 cases (12.2%), and in 3 cases (one 65, one 67, and one 94-year-old patient), it was described as a sole underlying disease. Pneumonia, as an acute symptom, was observed in 8 cases (4.6%), combined with respiratory failure, COPD, and pulmonary embolism, only one patient of which was younger than 65 years old, more precisely a 42-year-old female with Huntington’s chorea.

**Fig. 3:**
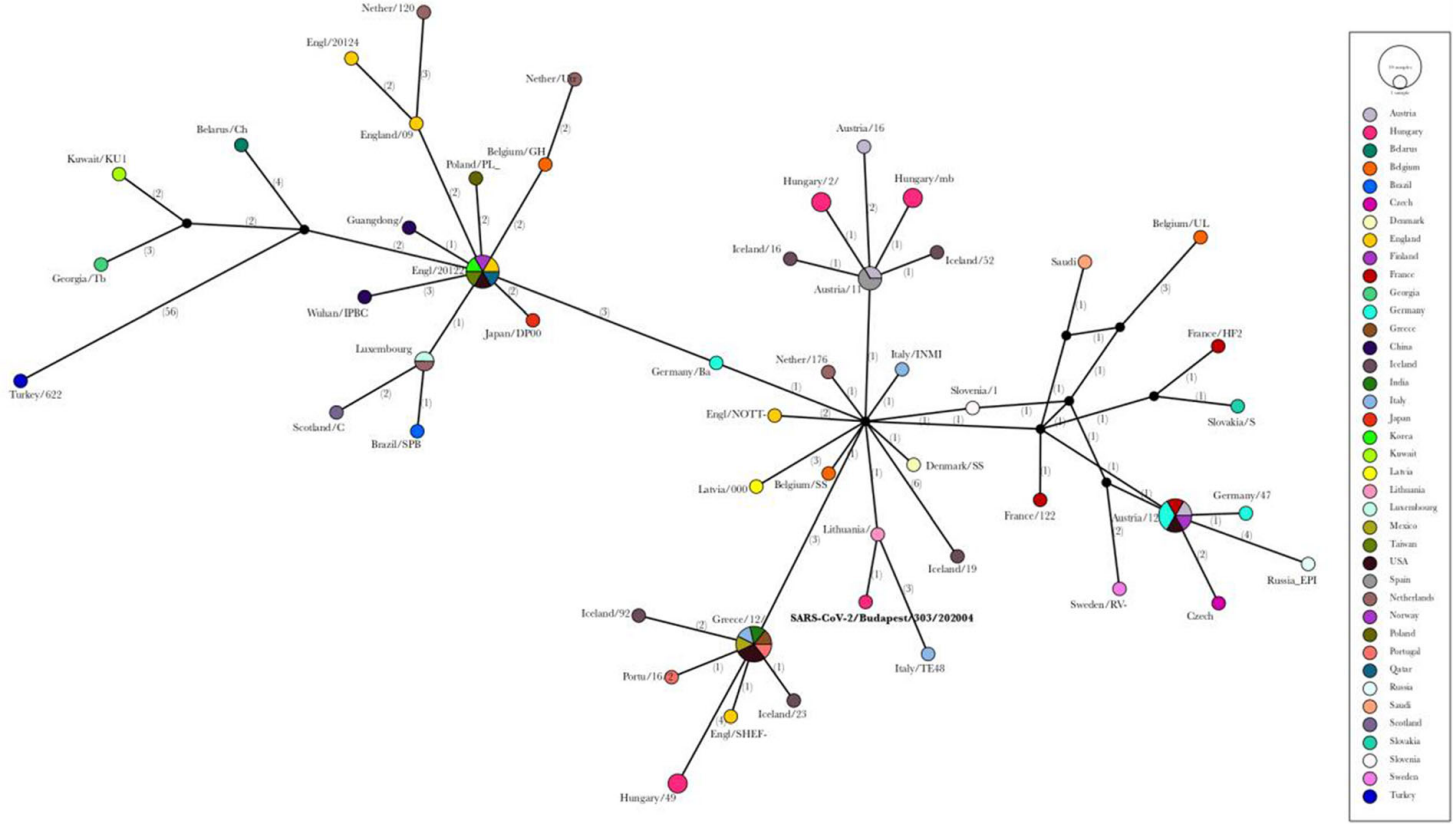
Median-joining network of 70 haplotypes mainly from Europe (4 out of this dataset is representative from Hungary). The sequence presented in this manuscript (SARS-CoV-2/Budapest/303/202004) is highlighted by bold text. Each circle is a representative of a different sequence type in which the size of the circle relates to the number of sequences. The color of the circles refers to the country of origin. The number of single-nucleotide polymorphisms is indicated with numbers in brackets

Sequence analysis resulted in the separate clustering of Hungarian haplotypes. The four Hungarian sequences separated into three different European haplotype clades. The nursing home sequence grouped with Lithuanian origin, separately from the other known in-country samples (Fig. 3).

Based on official data, a significant proportion (~ 11%, 198 individuals) of COVID-19 cases were related to social homes. Among these, eight individuals succumbed since the onset of the coronavirus outbreak. Altogether, 11 institutes have been affected thus far, all of them nursing home adult individuals (no child care institutes). Much of the control efforts were focused upon two institutes in the capital city (Budapest) in which more than 80 cases were found, while in the other institutes, the number of cases was less than 20, as of 18 April 2020.

## Discussion/conclusion

Based on available information to date and clinical expertise, data originated from Hungary are consistent with the global statements, in which most of the individuals who succumbed and were afflicted with confirmed COVID-19 infection in Hungary had at least one underlying chronic disease. According to the CDC reports, higher-risk group includes individuals with chronic lung disease or moderate to severe asthma, individuals who have serious heart conditions, individuals who are immunocompromised (e.g., as a consequence of cancer treatment, tobacco use, bone marrow or organ transplantation, immune deficiencies, poorly controlled HIV or AIDS, and prolonged use of corticosteroids and other immune weakening medications), individuals with severe obesity (body mass index [BMI] of 40 or higher), individuals with diabetes, with chronic kidney disease undergoing dialysis, or even with liver disease (CDC). Although in our data, cardiovascular disorders and diseases dominated, while chronic lung disease was present in negligible proportion, the same content of the above list mentioned by CDC was observed in the Hungarian fatalities as well, a vast part of individuals with multiple comorbidity, which is not surprising among the elderly. A recently published network-based analysis studied host-pathogen interactions (including tissue specificity), the molecular mechanisms of the infection and comorbidities as well, found that SARS-CoV-2 disease module does not directly overlap with any major underlying disease module; however, diseases closest to the COVID-19 proteins include several cardiovascular diseases and cancer, whose comorbidity in COVID-19 patients is well documented (Gysi et al. 2020).

Based on our sequence analysis, multiple introductory events are indicated from different European sources. The separate clustering and lack of connection between the Hungarian sequences most likely refer to the lack of sequence data. Thereby, the exact source of the nursing home sequence is hard to identify at this stage. However, it does not seem to be part of any abundant European sequence clades and is related at high extent to the Lithuanian sequence. More sequence data from Hungary and more widely from throughout Europe may reveal the exact origin and clustering level of this specific sequence.

Nursing homes play an important role as major clustering hotspots of the epidemic, and this is repeatedly reported by various media sources worldwide. Although published official data regarding nursing homes are only partially available in European countries, according to the report of the London School of Economics, snapshot data from varying official sources shows that nearly half of all COVID-19 deaths (ranging from 42 to 57%) appear to be happening in nursing homes, and at least in multiple European countries (e.g., Italy, Spain, France, Ireland, and Belgium) (https://www.theguardian.com/world/2020/apr/13/half-of-coronavirus-deaths-happen-in-care-homes-data-from-eu-suggests). Despite the limitations of our current knowledge, it is clear that these institutes demand special focus. In an effort to slow down the course of the epidemic, Hungary applied mitigation measures such as promoting social distancing and staying home, in order to reduce the transmission rate and shield the most susceptible elderly population, thereby preventing an over-burden to the health system. Furthermore, Hungary also applied a specific opening hours regime, ensuring daily between 0900 and 1200 h, only those above the age of 65 years are permitted to shop at grocery stores, markets, pharmacies, and drugstores. Consequently, seniors are not allowed to shop beyond this time frame. To our knowledge, this approach is unique and has been neither suggested nor implemented elsewhere.

Isolating the elderly may reduce transmission among this vulnerable social and age group, which is most important to reduce the devastating effect upon the nursing home hotspot emergence. This isolation may be applied as a mitigation effort focusing on the isolation of infected individuals, or more importantly, control measures should be applied to avoid the emergence of SARS-CoV-2 among these communities. Our results further emphasize the recent WHO statement regarding the vulnerability of seniors and especially the high risk of COVID-19 emergence in nursing and social homes (WHO 2020).
